# Use of a child health surveillance instrument focusing on growth. A cross-sectional study

**DOI:** 10.1590/1516-3180.2016.0345120617

**Published:** 2017-11-06

**Authors:** Erika Morganna Neves de Araujo, Marcia Teles de Oliveira Gouveia, Dixis Figueroa Pedraza

**Affiliations:** I Master’s Student, Nursing, Postgraduate Public Health Program, Universidade Estadual da Paraíba (UEPB), Campina Grande (PB), Brazil.; II PhD. Titular Professor, Postgraduate Nursing Program, Universidade Federal do Piauí (UFPI), Teresina (PI), Brazil.; III PhD. Titular Professor, Postgraduate Public Health Program, Universidade Estadual da Paraíba (UEPB), Campina Grande (PB), Brazil.

**Keywords:** Primary health care, Child care, Growth and development, Public health surveillance, Health records, personal

## Abstract

**BACKGROUND:**

Proper use of a child health handbook is an important indicator of the quality of care provided to children at healthcare services. This study aimed to evaluate the use of child health surveillance tool (by health professionals?), especially focusing on growth.

**DESIGN AND SETTING:**

Cross-sectional study carried out in the context of the Family Health Strategy in two municipalities in Paraíba, Brazil.

**METHODS:**

Three hundred and twenty-one children under five years of age from areas covered by health workers were included in the study. Mothers answered a questionnaire asking for information on sociodemographic characteristics. Growth charts, records of iron and vitamin A supplementation and notes on immunization schedules registered in the instrument were analyzed. In the case of children for whom the third version of the child health handbook was used, the association between completion of this handbook and sociodemographic characteristics was analyzed.

**RESULTS:**

All the parameters studied showed high frequencies of inadequate data entry, ranging from 41.1% for the weight-versus- age chart to 95.3% for the body mass index-versus-age chart. Higher frequency of inadequate data entry was found among children aged 25 months and over and among those living in areas of these municipalities with minimal numbers of professionals in the healthcare teams.

**CONCLUSIONS:**

The use of a child health handbook to monitor children’s growth in the municipalities studied appeared to be faulty. Data entry to this instrument was better at locations with larger healthcare teams.

## INTRODUCTION

Healthcare for children comprises development of health promotion and preventive care actions through highly qualified assistance, in order to contribute to adequate child growth and development, and to maintenance of health and quality of life.^1^ Primary healthcare services are the preferred gateway to the system, and links from this level to higher levels of care need to be established so as to offer comprehensive healthcare.^2^^,^^3^ From this perspective, child health handbooks, established in 2005 to replace the child card, stand out as an integral surveillance tool for children’s health, with periodic recording of anthropometric data in charts.^4^^,^^5^^,^^6^ At present, these handbooks offer the possibility of monitoring the evolution of weight, head circumference, height and body mass index, according to age groups, using curves developed by the World Health Organization for children up to the age of 10 years.^5^^,^^6^^,^^7^

Use of a specific instrument for monitoring children’s health is not exclusive to Brazil. Other countries such as the United Kingdom, Sweden, Greece, Portugal, France, Canada, Indonesia, Japan, Australia, New Zealand and the United States (some states) also use similar technologies, thus showing the relevance of such tools.^8^ In Indonesia, for example, this instrument has been the principal means of keeping health records since 2004 and its use has been correlated with better child immunization coverage.^9^

Proper use of a child health handbook, with complete and correct recording of information, not only is essential to the dialogue between healthcare professionals and users regarding its content but also can result in adherence and appreciation of the instrument by families, as well as shared responsibility for actions.^10^^,^^11^ With handbook data at hand, both parents and healthcare professionals have the opportunity to monitor the entire process of children’s growth and development, thus enabling early identification of health problems such as malnutrition and delayed growth, so that these do not become irreversible.^4^^,^^7^ Thus, use of a handbook is an important indicator of the quality of care provided to children at healthcare services.^5^^,^^11^ However, studies on this topic have noted that a variety of circumstantial difficulties may exist and have emphasized the need for further research.^7^^,^^11^^,^^12^

The objective of the present study was to evaluate the use of child health surveillance, especially focusing on growth.

## METHODS

This was a cross-sectional study carried out in two municipalities in the state of Paraíba. These municipalities were chosen based on their similarities regarding factors such as geographical location (metropolitan region of the state capital, with access to the service network available in the city), degree of urbanization (almost 100%), sociodemographic indicators, economic resources and tradition regarding organization of primary healthcare services (the “family health strategy” covers nearly 100% of the population). Municipality 1 has a population of 57,944 inhabitants, of whom 4,596 are children under five years of age. This municipality has a healthcare system composed of 19 family health strategy teams. Municipality 2 has a population of 99,716 inhabitants, of whom 7,862 are children under five years of age. Its healthcare system is composed of 28 family health strategy teams. There were more healthcare professionals (general physicians and pediatricians, nurses and nutritionists) for every 1,000 inhabitants, within the Brazilian National Health System (Sistema Único de Saúde, SUS), in municipality 1 than in municipality 2 at the time of this study, and the family health strategy teams are larger in municipality 1, with inclusion of nutritionists.

Children under five years of age were the study population. For the present study, a convenience sample was used, obtained from the sample of a larger study of which this formed part, which had the aim of evaluating healthcare service users’ perceptions about the quality of children’s health care. Parameters obtained from another study evaluating the quality of healthcare offered to children within the family health strategy, according to patients’ point of view, in the city of Montes Claros (Minas Gerais),^13^ were used to estimate the sample size in the major study.

In each municipality, nine family health strategy teams were randomly selected to represent at least one third of the total number of teams. Each team contributed 18 children on average. For the total sample, two criteria were followed:

1. An intentional sample of all nursing services that were part of routine visits on the day of data collection, on a typical working day; and
2. Selection of 18 out of all the children referred to healthcare services by community health workers, according to their work routine, to be included in the study based on the number of children intentionally sampled.

At the end of the data collection period, the sample consisted of 321 children under five years old, of whom 153 were from municipality 1 and 168 from municipality 2. Of this total, five children (one from municipality 1 and four from municipality 2) attended the healthcare unit without taking their handbook or card. Therefore, there were 316 evaluations for this study.

Data collection was carried out in the healthcare units between July and December 2014. The field team was composed of healthcare professionals and students who had had previous experience of fieldwork, and the team was supervised by a trained professional. The quality control for the study included: training and standardizing of interviewers, building an instruction manual and carrying out a pilot study in the city of Campina Grande, Paraíba.

The data collection included application of a questionnaire to the children’s mothers to obtain information on sociodemographic characteristics (child’s sex and age; mother’s age, employment and education level; number of people in the household; benefits received through the family grant program; and family income). The child’s age was calculated as the difference between the consultation date and the child’s date of birth. The municipality was also considered among the independent variables.

In order to assess the use of the child health surveillance tool by the health professionals, the interviewers analyzed the version that the interviewees had (use of the third version of the child health handbook was considered appropriate) and the records relating to growth (head circumference for age according to the head circumference-versus-age chart; weight for age according to the weight-versus-age chart; height for age according to the height-versus-age chart; and body mass index [BMI] for age according to the BMI-versus-age chart); supplementation (with iron according to the corresponding annotation table and with vitamin A according to the corresponding annotation table or the space for recording the vaccines); and vaccination (completeness of the vaccination table according to the space destined for this purpose).

**Figure 1.**
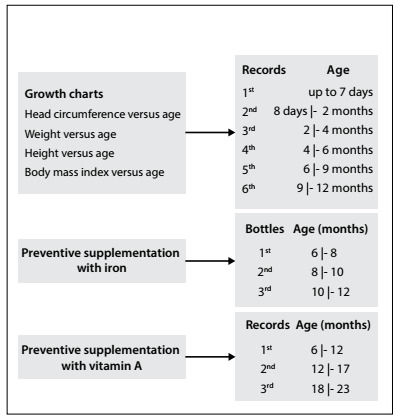
Adequacy parameters considered in determining the adequacy of records in growth charts and in charts on preventive iron and vitamin A supplementation, according to the child’s age.

The records were considered adequate when they were in accordance with the recommended standards, according to the child’s age at the time of the survey. The following recommendations established by the Ministry of Health were used: growth charts in accordance with the minimum number of visits indicated;^14^ preventive supplementation with iron in accordance with the national iron supplementation program;^15^ preventive supplementation with vitamin A in accordance with the national vitamin A supplementation program^16^ (Figure 1) and vaccination in accordance with the surveillance instrument itself. The height-versus-age curve was verified among children who had the second and third versions of the handbook. The BMI-versus-age curve was verified among the children who had the third version of the handbook. Supplementation with iron was verified in the annotation table for preventive iron and vitamin A supplementation. Vitamin A supplementation was verified in the annotation table for preventive iron and vitamin A supplementation or in the annotation table for vaccines.

In the case of children who had the proper instrument at hand, i.e. the third version of the child health handbook (n = 259), the completion of the instrument was classified as adequate or inadequate. To this end, a scoring system that allowed comparisons between different locations and over time was used.^17^ In order to calculate the score, a value of one was attributed for items correctly entered and zero for items incorrectly entered, and the total score was expressed as the sum of the values for these items. Thus, variation from 0 to 7 points was accepted, such that values closer to 7 indicated better completion. A score greater than or equal to four points (57.1% of the items correctly filled out) was established as the cutoff for adequate completion. This cutoff point was based on the statistical distribution of scores, given that no information on this cutoff point was encountered in the literature.

Data were organized in spreadsheets and double-entered. The validate application of the Epi Info software, version 3.3.2, was used to analyze data consistency, thus generating the final database used in the statistical analysis.

Estimates of prevalence ratios (PR) and their respective 95% confidence intervals (CI) were used to analyze the association between completing the third version of the child health handbook and sociodemographic characteristics. For adjustment of confounding factors, we used Poisson regression with robust variance adjustment. Variables that were associated with the level of 25% (P < 0.25) in bivariate analysis were included in multivariate analysis, in which P values according to the chi-square test were considered.

In all statistical analyses, the significance level accepted was 5%. Analyses were performed using the Statistical Package for the Social Sciences (SPSS) software, version 13.0.

This study was approved by the Ethics Committee of the State University of Paraíba, under protocol number 19689613.3.0000.5187.

## RESULTS

Among the 321 children studied, the majority (75.1%) were under 25 months of age. A high frequency of households with four or more people (67.0%) was observed. The vulnerability of the population was also evident in the proportion of the families enrolled in the family grant program (59.8%) and the proportion with family income below two minimum monthly wages (27.8%) (Table 1).

**Table 1.**
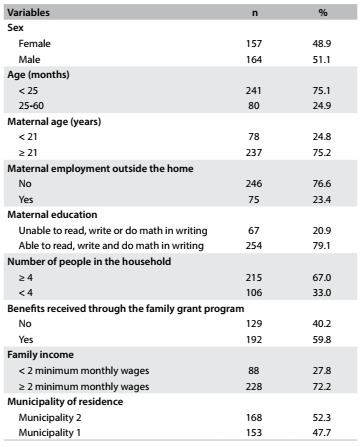
Sociodemographic characteristics of children under five years of age assisted by healthcare teams within the family health strategy in two municipalities in the state of Paraíba, 2014

Among the children who had the instrument at hand (316), 18% had inadequate versions (i.e. out-of-date versions). Regarding completion, all the parameters studied showed high frequencies of inadequate data entry, ranging from 41.1% for completion of the weight-versus-age chart to 95.3% for completion of the BMI-versus-age chart (Table 2).

**Table 2.**
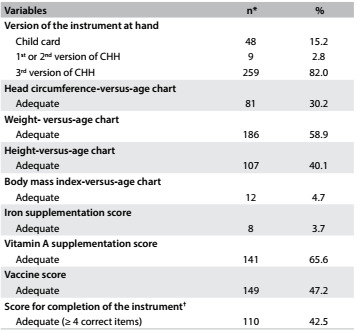
Child health surveillance instrument: version at hand and level of completion among children under five years of age who were treated by healthcare teams within the family health strategy in two municipalities in the state of Paraíba, 2014

CHH = child health handbook; *values that differ from the total were due to the version of the instrument used and/or the child’s age; ^†^completion level among children who had the third version of the child health handbook.

Table 3 presents the analyses on the associations between sociodemographic characteristics and completion of the child health handbook. The results show that there were statistically significant differences regarding the variables of child’s age and municipality of residence.

**Table 3.**
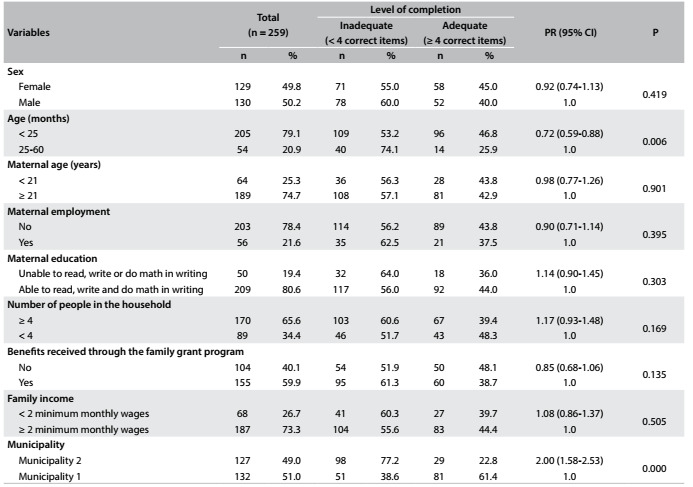
Association between level of completion of data entry in the child health handbook and sociodemographic characteristics of children under five years of age who were treated by healthcare teams within the family health strategy in two municipalities in the state of Paraíba, 2014

P = P-values for chi-square test; PR (95% CI) = prevalence ratio (with 95% confidence interval).

Through fitting the Poisson regression model, as shown in Table 4, it was observed that being a child under 25 months of age was a protective factor against inadequate data entry in the handbook (PR = 0.65; 95% CI: 0.53-0.80). Furthermore, children living in municipality 2, in relation to those living in municipality 1, had a higher frequency of inadequate data entry (PR = 1.55; 95% CI: 1.33-1.78).

**Table 4.**
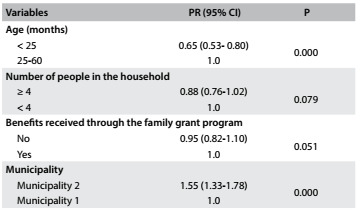
Adjusted association (Poisson regression) between inadequate completion of data entry in the child health handbook and sociodemographic characteristics of children under five years of age who were treated by healthcare teams within the family health strategy in two municipalities in the state of Paraíba, 2014

PR (95% CI) = prevalence ratio (with 95% confidence interval).

## DISCUSSION

It was observed that, even though nine years have elapsed since implementation of use of the child health handbook,^5^ 15.2% of the children in this study were still using the child card that preceded this. This result raises questions about the replacement of the card and the distribution of the handbook, as reported in a previous study.^18^

The results from the present study show problems relating to recording of growth charts, which have also been found in other places in Brazil.^5^^,^^18^^,^^19^^,^^20^ These findings suggest that there is non-compliance with the minimum number of consultations recommended by the Ministry of Health for the first five years of life,^14^ as also suspected by other researchers,^21^ and/or suggest that monitoring of growth has not yet received proper attention among healthcare professionals.^5^

The abovementioned situation, especially the disuse of height and body mass index charts, is a cause for concern because of the nutritional status of Brazilian children, which is characterized by coexistence of significant prevalence of growth retardation and overweight.^22^ These disorders, when untreated during childhood, can have irreversible consequences.^23^ This is why monitoring of growth is so essential.^7^

The higher percentage of completion of the weight-versus-age chart, compared with the other growth charts in this study, has been reported in a similar manner by other researchers, in Brazil^18^^,^^24^ and in other countries.^8^ This finding may be related to the fact that weight-versus-age curves have been used for a long time, i.e. since the implementation of the child card in 1984. It may also be associated with inclusion of new curves with concepts that are unfamiliar to professionals, such as representation as z scores.^6^

With regard to information on head circumference, the results from the municipalities studied here showed lower frequencies of correct data entry than was reported in other cities.^4^^,^^18^ These low percentages need to be analyzed with caution, because deviations from the recommended measurements may be associated with neurological diseases, such as microcephaly and hydrocephaly, which need better evaluation and referral.^15^

Like in previous studies,^20^ a low percentage of completion of the height-versus-age chart was seen in the present study. Non-availability of equipment and lack of professional training for making height measurements are possible factors relating to this problem.^25^ Systematic evaluation of the height-for-age index deserves greater attention at healthcare facilities because linear growth is an important expression of health and of the coverage of public policies.^26^

In this study, almost all BMI-versus-age charts were inadequately filled out. There is a lack of analyses in the literature in this regard.^23^ An evaluation of the medical records of children under two years of age in Cuiabá (Mato Grosso) showed that only 22.7% of the documents had registered BMI.^27^ Use of BMI is recommended from the time of birth onwards as a control measurement regarding overweight and adiposity, and as a predictor of obesity in adulthood.^23^

The greater adequacy of completion of data entry in the child health handbook among younger children that was observed in the present study has similarly been reported in previous investigations.^24^ This can possibly be explained by the higher frequency of scheduling of consultations during children’s first months of life, as a reflection of the greater biological risk that is typical of this extremely young age.^23^^,^^24^ Moreover, during the first year of life, children receive most of their vaccines, thus implying that there is a need to return to the healthcare service more often, which gives rise to opportunities for use of this instrument.^20^ It should be noted that the Poisson regression with robust variance that was used in the present study to obtain this result has been suggested as an appropriate method for cross-sectional designs with binary outcomes, which should be based on calculation of prevalence ratios.^28^

Considering the particular features of the municipalities analyzed, it is possible that the significant difference in the rate of inadequate completion of data entry in the child health handbook that was observed between these two municipalities was related to differences between them regarding the structure of larger teams and the training of professionals to work within primary healthcare and develop food and nutrition actions. Both of these municipalities had adequate availability of materials, inputs and equipment, including anthropometric instruments and handbooks. These conditions may favor interaction and minimize the influence of factors that hinder completion of the handbook, at least as far as food and nutrition issues are concerned. Experiences from other countries have shown that interactions among primary healthcare professionals promote improvement of growth monitoring and, in turn, the nutritional status of children.^29^ However, further research in the Brazilian context should be conducted to address the possible matters of controversy.

The need for caution in interpreting the findings from the present study has to be emphasized, given that the responsibility for using the child health surveillance instrument is not restricted to healthcare professionals but should be shared with families.^23^^,^^30^ Awareness needs to be raised among all parties involved regarding the importance of making proper use of the handbook and of effectively achieving its purpose.^5^

In interpreting the results from this study, some limitations should be considered. Firstly, it should be noted that data entry in the child health handbook was restricted only to some of the parameters included in the instrument. Furthermore, some factors relating to completion of the data entry in the handbook were not considered here, such as birth weight and other conditions inherent to child health, maternal care during the prenatal and postpartum period, the area of residence (urban or rural) and the parents’ marital status. Furthermore, because of the attempt to preserve the work routine through use of a convenience sample, the possibility of bias in selecting the sample of children may be presumed.

## CONCLUSIONS

Although the child health handbook is one of the most prominent aspects of public health policies in Brazil, the use of this instrument to monitor children’s growth within the family health strategy in the municipalities studied is flawed. The precariousness of the records suggests that there is a need to raise awareness about the importance of the handbook and of larger healthcare teams.
